# DISCRIMINATION ACROSS THE LIFE COURSE OF OLDER LONG-TERM RESIDENTS IN KOREA

**DOI:** 10.1093/geroni/igae098.4314

**Published:** 2024-12-31

**Authors:** Jasmyn Kim, Jinmoo Heo

**Affiliations:** Yonsei University, Seoul, Republic of Korea; Yonsei University, Seoul, Republic of Korea

## Abstract

Despite being one of the world’s most homogenous nations, South Korea’s (hereafter Korea) international population has grown significantly over the past few decades. As of 2022, approximately 2.5 million foreign citizens reside in Korea, comprising 5% of the total population and contributing to its evolving identity as a multicultural society. This demographic shift parallels Korea’s rapid socio-economic growth. However, foreign residents often encounter substantial challenges due to the persistent notion of a unified racial, ethnic, and cultural identity in Korean society, which can lead to cultural differences, social exclusion, stereotypes, and discrimination against those considered ‘others.’ The current qualitative case study explores the experiences of older long-term foreign residents from Western countries, focusing on discrimination across the life course. In-depth interviews were conducted with older international adults (mean age: 64.5) who had resided in Korea for over 3 years. Key findings revealed that despite experiencing alienation and exclusion from mainstream society, participants highlighted the importance of establishing social connections within both expatriate and local communities and reported a sense of belonging fostered through intercultural exchange, personal relationships and professional achievements. Although there is a growing emphasis on multiculturalism, prevailing cultural norms often hinder the complete integration of foreign residents into Korean society. This research sheds light on the complex power dynamics of ethnicity, social status, and discrimination, highlighting the need for more inclusive policies to better incorporate foreign residents in an increasingly globalized society.

